# Altered serum TNF-α and MCP-4 levels are associated with the pathophysiology of major depressive disorder: A case-control study results

**DOI:** 10.1371/journal.pone.0294288

**Published:** 2023-11-15

**Authors:** Jannatul Nayem, Rapty Sarker, A. S. M. Roknuzzaman, M. M. A. Shalahuddin Qusar, Sheikh Zahir Raihan, Md. Rabiul Islam, Zobaer Al Mahmud

**Affiliations:** 1 Department of Clinical Pharmacy and Pharmacology, Faculty of Pharmacy, University of Dhaka, Dhaka, Bangladesh; 2 Department of Pharmacy, University of Asia Pacific, Dhaka, Bangladesh; 3 Department of Psychiatry, Bangabandhu Sheikh Mujib Medical University, Shahabagh, Dhaka, Bangladesh; 4 School of Pharmacy, BRAC University, Dhaka, Bangladesh; Fukuoka University, JAPAN

## Abstract

**Background:**

Major Depressive Disorder (MDD) is a debilitating mental health condition with complex etiology, and recent research has focused on pro-inflammatory cytokines and chemokines as potential contributors to its pathogenesis. However, studies investigating the roles of TNF-α and MCP-4 in MDD within the Bangladeshi population are scarce. This study aimed to assess the association between serum TNF-α and MCP-4 levels and the severity of MDD, exploring their potential as risk indicators for MDD development.

**Methods:**

This case-control study enrolled 58 MDD patients from Bangabandhu Sheikh Mujib Medical University (BSMMU) Hospital, Dhaka, Bangladesh, alongside 30 age, sex, and BMI-matched healthy controls. MDD diagnosis followed DSM-5 criteria and disease severity using the 17-item Hamilton Depression Rating Scale (Ham-D). We measured serum TNF-α and MCP-4 levels using ELISA assays according to the supplied protocols.

**Results:**

The study revealed significantly elevated serum TNF-α levels in MDD patients (47±6.6 pg/ml, mean±SEM) compared to controls (28.06±1.07 pg/ml). These increased TNF-α levels positively correlated with Ham-D scores (Pearson’s r = 0.300, p = 0.038), suggesting a potential association between peripheral TNF-α levels and MDD pathology. Additionally, MDD patients exhibited significantly higher serum MCP-4 levels (70.49±6.45 pg/ml) than controls (40.21±4.08 pg/ml). However, serum MCP-4 levels showed a significant negative correlation (r = -0.270, P = 0.048) with Ham-D scores in MDD patients, indicating a more complex role for MCP-4 in MDD pathogenesis.

**Conclusion:**

This study highlights that Bangladeshi MDD patients exhibit heightened inflammatory and immune responses compared to controls, supporting the cytokine hypothesis in MDD pathogenesis. Serum TNF-α, but not MCP-4, shows promise as a potential biomarker for assessing the risk of MDD development, which could aid in early detection. Future investigations involving larger populations and longitudinal studies are essential to confirm the utility of these cytokines as biomarkers for MDD.

## 1. Introduction

Major Depressive Disorder (MDD), commonly termed depression, is a serious and prevalent mental health condition characterized by persistent feelings of sadness, hopelessness, and a lack of interest or pleasure in activities. It influences an individual’s emotional state, cognitive processes, and overall physical health, frequently hindering one’s ability to carry out everyday activities and diminishing their overall quality of life. The symptoms associated with MDD encompass a range of manifestations, such as reduced energy levels, alterations in sleep patterns, disruptions in eating, challenges in maintaining focus, and experiences of guilt or feelings of worthlessness [1, 2]. Individuals diagnosed with MDD may exhibit psychomotor agitation or retardation symptoms, resulting in either heightened restlessness or reduced motor activity [3]. There is considerable variation in the severity and duration of symptoms experienced by different individuals. Almost half of those diagnosed with depression have suicidal thoughts, whereas a smaller percentage, ranging from 10% to 15%, ultimately engage in suicidal behavior [4]. A significant proportion of individuals do not exhibit a positive response to antidepressant therapy, with approximately 50% failing to show improvement. Also, 20% of individuals continue to exhibit resistance to all available interventions [4]. Projections suggest that depression will become the second leading cause of global disability by 2030, ranking only below HIV in terms of its impact on population health [5, 6]. The etiology of MDD is multifaceted, encompassing genetic, biochemical, environmental, and psychological influences. MDD is a substantial issue in global health, with an approximate prevalence of 15% worldwide [7]. The available treatment modalities encompass psychotherapeutic interventions, such as cognitive-behavioral therapy (CBT) and interpersonal therapy (IPT), in addition to pharmacological approaches involving selective serotonin reuptake inhibitors (SSRIs) and serotonin-norepinephrine reuptake inhibitors (SNRIs). In instances of heightened severity, the utilization of electroconvulsive treatment (ECT) or transcranial magnetic stimulation (TMS) may be contemplated [8].

The etiology of MDD encompasses a complex interplay among genetic, neurological, and environmental components. The significance of neurotransmitter abnormalities, specifically within the serotonin, norepinephrine, and dopamine systems, has been highlighted in recent research [9]. The decreased levels of these neurotransmitters disrupt communication between brain regions involved in mood regulation, cognition, and emotion processing. The dysregulation of the hypothalamic-pituitary-adrenal (HPA) axis, which is responsible for responding to stress, is associated with increased levels of cortisol, leading to impacts on mood and physiological processes [10–12]. The presence of neuroinflammation, as shown by elevated levels of cytokines, has been observed in individuals with MDD and may impact neuroplasticity and the regulation of mood-related pathways. An increase in cytokines can affect neurotransmitter metabolism, the integrity of the blood-brain barrier, and the neuroplasticity processes and synaptic function [13]. Existing research suggests that people diagnosed with MDD frequently demonstrate increased concentrations of pro-inflammatory cytokines and disrupted levels of anti-inflammatory cytokines [14, 15]. The disruption of immunological signaling can potentially disturb the intricate equilibrium between pro-inflammatory and anti-inflammatory processes, which may impact brain circuits associated with mood regulation, cognition, and behavior [16]. The "inflammatory hypothesis" posits that persistent inflammation, induced by diverse stressors or environmental factors, may affect the onset and intensification of depression symptoms [12, 17].

The correlation between cytokines and chemokines with MDD reveals a significant connection between the immune system and mental well-being. The significance of cytokines, small protein molecules involved in immune responses, and chemokines, which drive immune cell migration and inflammation, in the pathophysiology of MDD has garnered significant interest in recent studies [14, 15]. The association between neuroinflammation and depressive symptoms suggests that chemokines may substantially impact the etiology and persistence of MDD. Consequently, a growing body of clinical research has documented the probable involvement of CC chemokines, such as monocyte chemoattractant protein (MCP)-1, macrophage inflammatory protein (MIP)-1α, MIP-1β, and CXC chemokines, including interleukin (IL)-8, in MDD [18, 19]. CC chemokines, such as MCP-1, MIP-1α, and MIP-1β, as well as CXC chemokines, including CXCL8, CXCL10, and CXCL4, exhibited a significant increase in MDD patients compared to the control group [18–21]. Furthermore, the research indicated that these elevated serum levels were significantly reduced after antidepressant treatments [15, 18], suggesting a potential association between these chemokines and MDD.

To understand the intricate pathophysiology of MDD, specific cytokines, and chemokines have garnered attention for their potential roles in shaping the disorder’s underlying mechanisms. Notably, Tumor Necrosis Factor-α (TNF-α) and MCP-4 have emerged as intriguing cytokines contributing to our comprehension of MDD’s intricate web. TNF-α, a pro-inflammatory cytokine, plays a multifaceted role in the pathophysiology of MDD [5, 22]. Researchers observed elevated levels of TNF-α in individuals with MDD, and its presence is associated with activation of the immune response. TNF-α’s influence extends to the nervous system, impacting neurotransmitter function, neuronal plasticity, and synaptic connectivity [5]. The cytokine’s pro-inflammatory effects may disturb the balance of neurotransmitters such as serotonin and dopamine, critical for mood regulation. Also, TNF-α can promote oxidative stress and contribute to neural cell damage, further implicating its role in the neurodegenerative aspects of MDD [23–30]. MCP-4, a chemokine, is also gaining recognition for its potential involvement in MDD [31, 32]. As a key player in immune cell recruitment and activation, MCP-4 can influence inflammation and immune response regulation. Altered levels of MCP-4 have been associated with depressive symptoms, suggesting its role in the intricate interplay between the immune system and mood regulation [32]. MCP-4 mediates the cross-talk between the peripheral and central immune systems by triggering the chemotaxis of monocytes and other immune cells, leading to the infiltration of monocytes and other immune cells from the peripheral system to inflammatory sites in the CNS [33]. So, MCP-4 mediates cell-to-cell communication and causes infiltration of inflammatory cells into the CNS, resulting in neuroinflammation [34]. Though these activities are sometimes beneficial for immune responses protecting the CNS, low and chronic neuro-inflammation causes disruption in neuronal circuits and several dopaminergic, adrenergic, or serotonergic pathways that ultimately lead to MDD [35]. Neuro-inflammation triggered by TNF-α or MCP-4-mediated infiltration of monocytes could also destroy glial cells, including oligodendrocytes, astrocytes, and microglia. Despite having the potential to regulate mood disorders, MCP-4 remains a poorly understood chemokine, and there is a scarcity of clinical studies evaluating the putative role of MCP-4 in MDD [32]. The associations of TNF-α and MCP-4 with MDD underscore the intricate interplay between immunological processes and mental health. Altered levels of these immune molecules in individuals with MDD suggest a possible contribution to the systemic inflammation often observed in the disorder. Elucidating the roles of TNF-α and MCP-4 in MDD will not only advance our understanding of the underlying mechanisms but also present opportunities for developing targeted interventions that address the immunological dimension of the disorder.

Despite considerable progress in understanding MDD, several significant knowledge gaps persist in this complex and multifaceted mental health condition. One issue revolves around the underlying neurobiological mechanisms of MDD [36, 37]. The precise intricacies of how neurotransmitter imbalances lead to the constellation of depressive symptoms remain elusive despite the role of altered neurotransmitters in the pathophysiology of depression. Additionally, the role of neuroinflammation and the immune system in MDD is an area of growing interest. However, the precise relationships between immune dysregulation, neural circuits, and mood disturbances require further elucidation [38–40]. The identification of reliable and objective biomarkers for MDD remains a challenge. While research has explored potential biomarkers in blood, cerebrospinal fluid, and neuroimaging data, a lack of consistent markers for diagnosis, prognosis, and treatment response hinders personalized approaches to MDD management [40–42]. As we don’t have any specific biomarkers for MDD, emerging evidence suggests that cytokines like TNF-α and chemokines like MCP-4 could hold potential candidates.

Our study aims to investigate the correlation between MDD and these potential biomarkers to bridge this knowledge gap. By examining the levels of TNF-α and MCP-4 in both MDD patients and healthy controls, we seek to uncover whether alterations in these immune molecules are associated with MDD. To address this objective, we have designed a comprehensive case-control study comparing the circulating levels of TNF-α and MCP-4 between individuals diagnosed with MDD and a matched group of healthy individuals. This study endeavors to shed light on the utility of TNF-α and MCP-4 as potential biomarkers for MDD, advancing our understanding of the complex interplay between immune dysregulation and depressive disorders and offering promising insights into improved diagnostic and therapeutic approaches.

## 2. Methods and materials

### 2.1 Study design

In this study, we conducted a case-control investigation, including 88 participants from MDD patients and healthy controls (HCs). We recruited MDD patients from the Department of Psychiatry of Bangabandhu Sheikh Mujib Medical University (BSMMU) in Bangladesh and HCs from various areas of Dhaka from October 1, 2022, to December 31, 2022. Our study focused on 18-to-60-year-old male and female adults. We included 58 MDD patients and 30 HCs matched with their sexes and ages. Experienced psychiatrists performed thorough clinical interviews of participants following DSM-5 criteria to ensure the accuracy of clinical evaluations. We considered cases if depressive symptoms persisted for at least two weeks. Exclusion criteria included participants with a history of cardiac, hepatic, kidney, inflammatory, or severe somatic disorders and those who had consumed alcohol or illicit substances within the previous six months. In addition, participants abstained from taking any medications, including antidepressants and antipsychotics, for at least one week before the evaluation to prevent potential interference with serum TNF-α and MCP-4 levels. Also precluded from the study were pregnant women and those with other underlying medical conditions. Using a pre-structured questionnaire, we gathered sociodemographic data from each participant in the study.

### 2.2 Collection, processing, and storage of blood samples

We collected 5 ml of blood samples from each MDD patient and HCs for laboratory analysis. The blood was allowed to cot for one hour at room temperature. To separate serum from whole blood, we centrifuged collected blood samples at 1000 g for 15 minutes. The serum was then placed in Eppendorf containers and frozen at -80°C. To determine the serum levels of TNF-α and MCP-4, we used commercially available human TNF-α and MCP-4 ELISA kits, respectively (Boster Bio, USA).

### 2.3 Sample analysis

We followed the manufacturer’s instructions throughout the complete procedure for analyzing samples. At first, we dispensed 100μl of serum sample and standard solution into the corresponding wells of a 96-well microplate. After covering the plate with a sealer, we incubated it at 37°C for 90 minutes. Then, we discarded the liquids from the wells and added 100μl of the respective cytokine’s detection antibody to specific wells with thorough mixing. After resealing the plate, we incubated it at 37°C for 60 minutes. Following this, we aspirated the contents of each well and rinsed three times with 300μl of wash buffer. Next, 100μl of the avidin-biotin-peroxidase complex was added to each well, and the plates were incubated at 37°C for 30 minutes. After discarding the liquids, each plate was washed with 300μl of wash buffer five times. To complete the procedure, we added 90μl of color-developing agent to each well, followed by 100μl of stop solution. Finally, we promptly measured absorbance at 450 nm, calculating serum TNF-α and MCP-4 concentrations in pg/ml.

### 2.4 Statistical analysis

We used GraphPad Prism version 5.0b (GraphPad, San Diego, USA) and SPSS statistical software version 25.0 (IBM Corp., Armonk, NY) for data analysis. To summarize the demographic and clinical characteristics of the study population, descriptive statistics were used. The data were presented as the mean ± standard error mean (SEM). We applied an unpaired two-tailed student’s t-test to determine the statistical significance of differences in age, sex, BMI, and other parameters between the patient and HCs. We used dot-plot graphs to compare study parameters between the groups. Also, we compared the serum levels of the relevant cytokines between MDD patients and HCs using a two-tailed, unpaired Student’s t-test. The correlations between cytokine levels and disease severity (Ham-D scores) were analyzed using Pearson’s correlation coefficient. To distinguish MDD patients from HCs, we analyzed TNF-α and MCP-4 levels using Receiver Operating Characteristics (ROC) curves. We computed diagnostic precision metrics, including sensitivity, specificity, and positive/negative predictive values. In all cases, we regarded p<0.05 to be statistically significant.

### 2.5 Ethical consideration

The research protocol was approved by the Ethical Review Committee of the Faculty of Pharmacy, University of Dhaka, Bangladesh (Ref. No. Fa. Ph. E/009-A/22). The objectives of the study were communicated to the participants, and written consent was obtained from each participant. We conducted this investigation in accordance with the Helsinki Declaration’s guiding principles.

## 3. Results

### 3.1 Socio-demographic and clinical characteristics of study population

The socio-demographic characteristics of the study subjects are presented in Table 1. MDD patients and HCs were very similar by age, percentage of males and females, and BMI values. The average age for recruited MDD patients was 30.57±1.33 years, while that for HCs was 30.77±1.75 years, and there was no significant variation between them (p = 0.930). Similar percentages of males (31.04% and 30.00%) and females (68.96% and 70.00%) were recruited in patients and HCs, respectively. Similarly, there was no statistically significant (p = 0.261) variation in BMI values between patients (23.09±0.48 kg/m^2^) and HCs (24.22±1.04 kg/m^2^). There were no significant variations for other socio-demographic parameters between the groups (Table 1). The clinical characteristics and laboratory findings of the study population are presented in Table 2. Ham-D scores were determined to express the severity of the disease. MDD patients displayed significantly higher Ham-D scores (17.88±0.73) than HCs (0.66±0.21). However, female (19.23±0.87) MDD patients displayed significantly higher Ham-D scores than their male counterparts (14.89±1.07).

**Table 1:** Sociodemographic characteristics of the study population.

Parameters	MDD patients (*n* = 58) Mean ± SEM	Healthy controls (*n* = 30) Mean ± SEM	p value
Age in years	30.57±1.33	30.77±1.75	0.930
18–25	19 (32.76%)	10 (33.33%)	
26–35	25 (41.10%)	11 (36.67%)	
36–45	9 (15.52%)	7 (23.33%)	
46–60	5 (8.62%)	2 (6.67%)	
Sex			0.641
Male	18 (31.04%)	9 (30.00%)	
Female	40 (68.96%)	21(70.00%)	
Marital status			0.382
Married	38 (65.52%)	19 (63.33%)	
Unmarried	20 (34.48%)	11 (36.67%)	
BMI (kg/m^2^)	23.09±0.48	24.22±1.04	0.261
Below 18.5 (CED)	06 (10.35%)	0 (0.00%)	
18.5–25 (normal)	39 (67.24%)	15 (50.00%)	
Above 25 (obese)	13 (22.41%)	15 (50.00%)	
Education level			0.162
Illiterate	1 (1.73%)	3 (10.00%)	
Primary level	12 (20.69%)	4 (13.34%)	
Secondary level	13 (22.41%)	3 (10.00%)	
Higher Secondary level	15 (25.86%)	7 (23.33%)	
Graduate and above	17 (29.31%)	13 (43.33%)	
Occupation			0.541
Housewife	26 (44.83%)	11 (36.67%)	
Service	13 (22.41%)	8 (26.67%)	
Unemployed	8 (13.79%)	4 (13.33%)	
Student	0 (0.00%)	1 (3.33%)	
Others	11 (18.97%)	6 (20.00%)	
Economic status			0.829
High	4 (6.89%)	3 (10.00%)	
Medium	35 (60.35%)	18 (60.00%)	
Low	19 (32.76%)	9 (30.00%)	
Smoking history			0.104
Nonsmoker	53 (91.38%)	30 (100.00%)	
Smoker	5 (8.62%)	0 (0.00%)	
Residence area			0.689
Rural	18 (31.03%)	9 (30.00%)	
Urban	40 (68.97%)	21(70.00%)	
Family history of MDD			0.084
Yes	7 (12.07%)	0 (0.00%)	
No	51 (87.93%)	30 (100.00%)	

Abbreviations: BMI, body mass index; CED, chronic energy deficiency; MDD, major depressive disorder; SEM, standard error mean. p<0.05 is considered to be statistically significant.

**Table 2:** Clinical characteristics and laboratory parameters of MDD patients and healthy controls.

Cytokine	MDD patients (*n* = 58) Mean ± SEM	Healthy controls (*n* = 30) Mean ± SEM	p value
Ham‐D scores	17.88±0.73	0.66±0.21	<0.001
Male	14.89±1.07	0.55±0.24	
Female	19.23±0.87	0.71±0.28	
Serum TNF-α (pg/ml)	47.00±6.60	28.06± 1.07	0.043
Male	45.52±5.43	28.63±2.51	
Female	48.87±9.40	27.76±1.03	
Serum MCP-4 (pg/ml)	70.49±6.45	40.21±4.09	0.002
Male	69.10±7.92	45.96±8.30	
Female	71.17±8.86	37.80±4.68	

Data are expressed as mean±SEM and analyzed by unpaired two-tailed student’s t test for determining the level of significance between mean difference between patients and control groups. P< 0.05 is considered to be statistically significant. Ham-D, 17-item Hamilton depression rating scale; MDD, major depressive disorder; SEM, standard error mean; MCP-4, monocyte chemoattractant protein-4; TNF-α, tumor necrosis factor-alpha.

### 3.2 Laboratory findings

**Fig 1:**
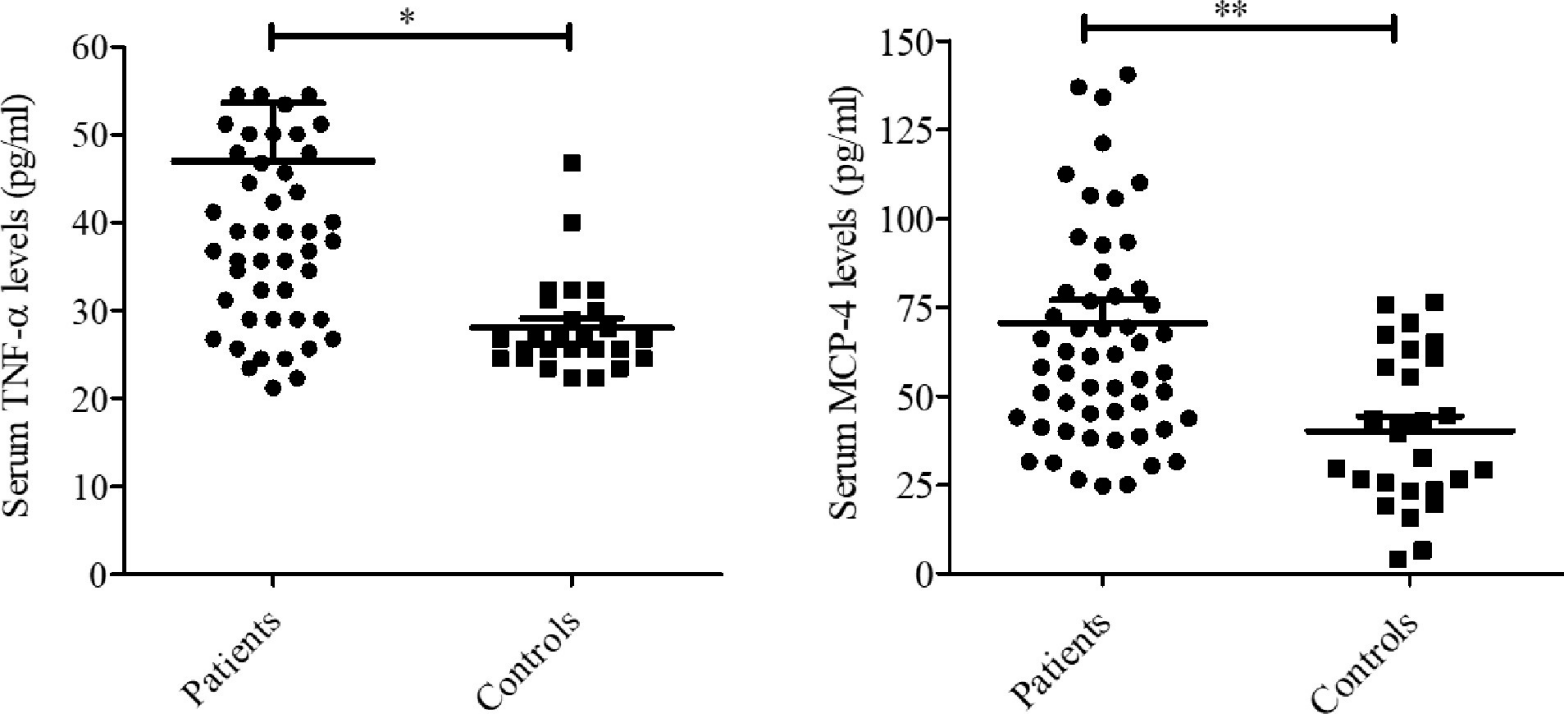
Differences of serum TNF-α and MCP-4 levels between MDD patients and healthy controls.

In our case-control study, we found significantly (p = 0.043) higher serum levels of TNF-α in MDD patients (47.00±6.60 pg/ml) compared to HCs (28.06±1.07 pg/ml) that indicates TNF-α might be associated with the pathophysiology of MDD (Table 2 and Fig 1). Another finding was that similar levels of TNF-α were observed in male and female MDD patients (Table 2) which implies no significant variation of inflammatory response in MDD between males and females of Bangladeshi origin. A significantly (p = 0.002) higher serum MCP-4 levels were also observed in MDD patients (70.49±6.45 pg/ml) compared to HCs (40.21±4.09 pg/ml) indicating that this chemokine might be associated with the disease pathogenesis (Table 2 and Fig 1). As for TNF-α level, a similar magnitude of serum MCP-4 levels was observed in male and female MDD patients (Table 2).

### 3.3 Association between TNF-α and MCP-4 with MDD severity

**Fig 2:**
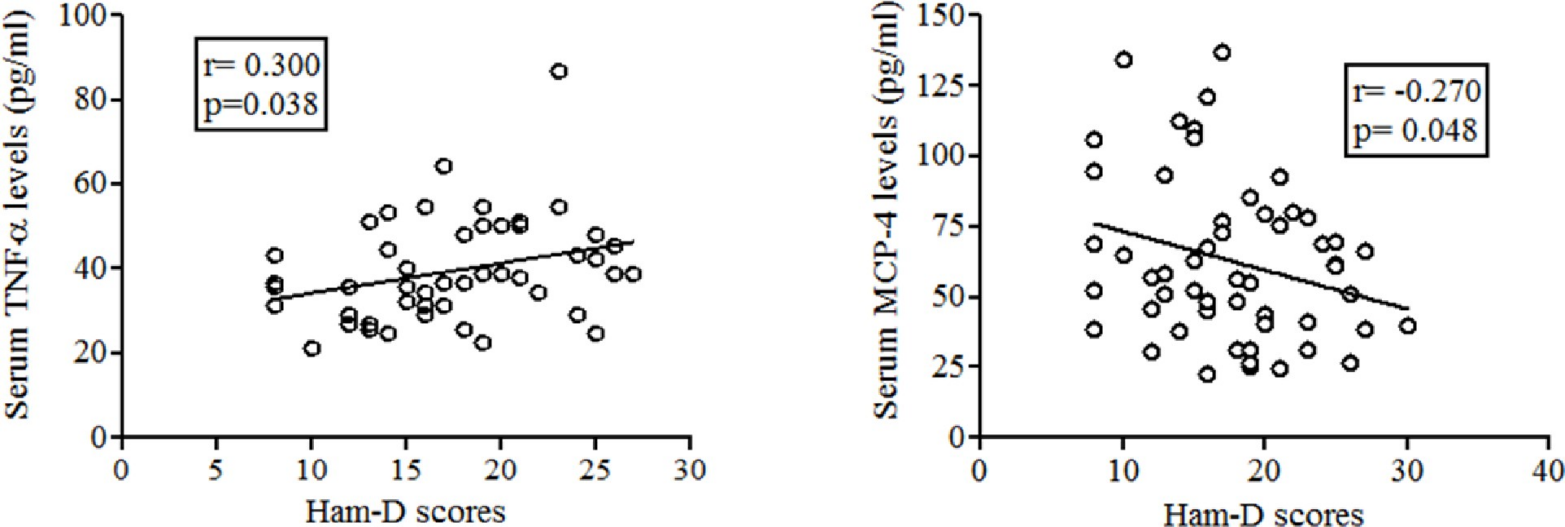
Correlation between serum TNF-α and MCP-4 levels with the severity scores among the MDD patients.

We observed that serum TNF-α levels are significantly and positively (Pearson’s correlation coefficient, r = 0.300, p = 0.038) (Fig 2) correlated with the Ham-D scores in MDD patients implicating a moderate strength of association between the disease severity and TNF-α serum levels. Contrary to the results obtained for TNF-α, Pearson’s correlation analysis showed that serum MCP-4 levels were significantly and negatively correlated (r = -0.270, p = 0.048) (Fig 2) with Ham-D scores in MDD patients suggesting that higher MCP-4 levels are associated with lower severity of the disease and lower MCP-4 levels are linked to the higher disease severity.

### 3.4 Receiver operating characteristics analysis

**Fig 3:**
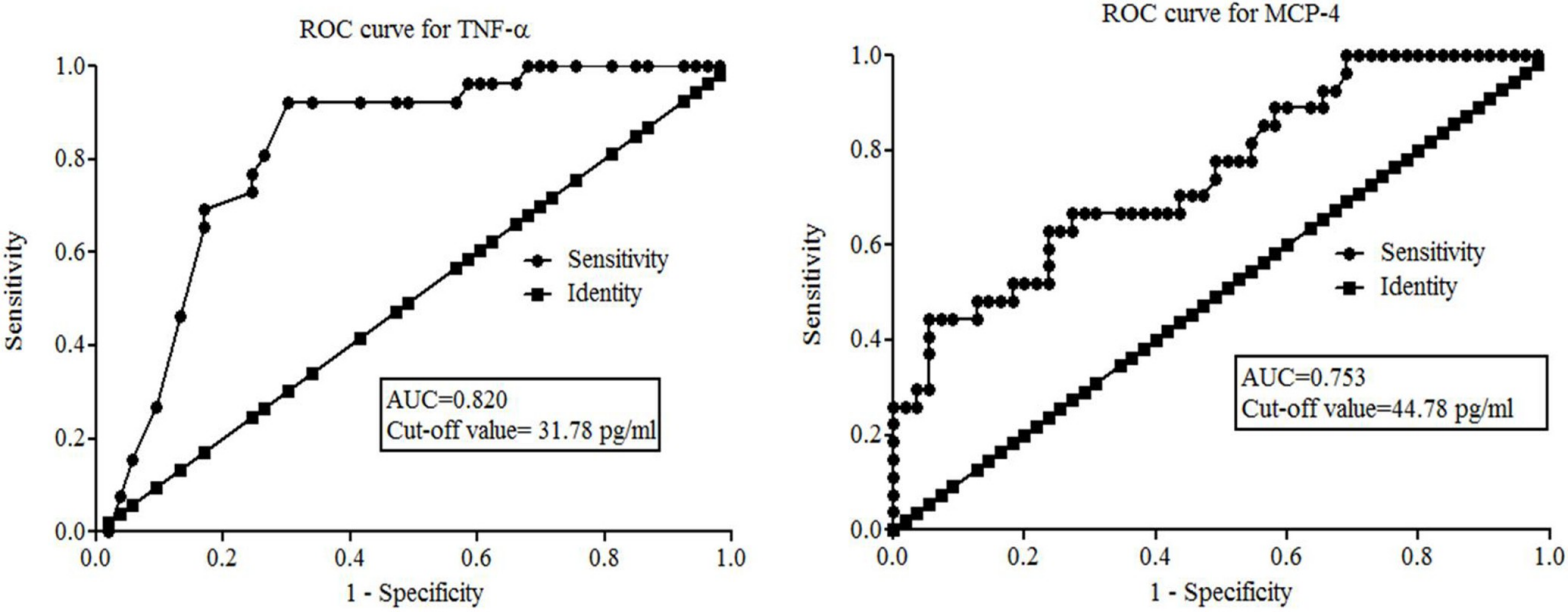
Receiver Operating Characteristic (ROC) curve for serum TNF-α and MCP-4 levels in discriminating MDD patients from healthy controls.

We performed Receiver Operating Characteristics (ROC) analysis to evaluate the diagnostic efficacy or accuracy of serum TNF-α and MCP-4 levels in differentiating patients with MDD from HCs. ROC analysis revealed that serum TNF-α levels displayed good diagnostic value with an area under the curve (AUC) of 0.820 (CI: 0.726 to 0.914, p<0.001) with 80.77% sensitivity and 73.58% specificity at a cut-off point value of 31.78 pg/ml. Also, serum MCP-4 levels showed good diagnostic performance with AUC value of 0.753 (CI: 0.642 to 0.864, p<0.001) and percentages of sensitivity (66.67%) and specificity (72.73%) at an optimal cut-off value of 44.78 pg/ml (Table 3 and Fig 3).

**Table 3:** ROC analysis for serum levels of TNF-α and MCP-4 as discriminators between MDD patients and healthy controls.

Cytokine	Cut-off value (pg/ml)	AUC	95% CI		Sensitivity (%)	Specificity (%)	PPV (%)	NPV (%)	p value
			Lower limit	Upper limit					
TNF-α	31.78	0.820	0.726	0.914	80.77	73.58	88.64	60.00	<0.001
MCP-4	44.78	0.753	0.642	0.864	66.67	72.73	81.63	54.54	<0.001

ROC, Receiver operating characteristics curve; CI, confidence interval; AUC, area under the ROC curve; PPV, positive predictive value; NPV, negative predictive value.

## 4. Discussion

We observed serum TNF-α level was significantly higher in MDD patients compared to HCs, and these elevated serum levels were positively and moderately correlated with disease severity, implicating that peripheral levels of TNF-α might be associated with MDD disease pathogenesis. We observed a 1.67-fold increase in serum TNF-α levels in MDD patients compared to HCs. Xu et al. (2023) also observed a 1.73-fold increase in serum TNF-α levels in MDD patients (4.63 ± 0.39 pg/ml) compared to HCs (2.67 ± 0.11 pg/ml) [18]. Consistent with our result, Fan et al. (2017) also reported a two-fold elevation in serum TNF-α levels in MDD patients (4.65±1.58 pg/ml) compared to HCs (2.24±1.58) [43]. However, the 12.5-fold higher serum TNF-α levels observed in Bangladeshi HCs compared to that of a Chinese cohort might reflect the higher inflammatory status of the Bangladeshi population that might be due to the ethnic variation in cytokine expression profile among them. Yao et al. (2020) also recruited a similar number of patients (n = 57) and HCs (n = 30) in their study and observed a 2.20-fold increase in TNF-α levels in MDD patients compared to HCs [44]. Several researchers also observed elevated serum levels of TNF-α in MDD patients compared to HCs [45–52]. In contrast to our findings, Farid et al. (2007) and Einvik et al. (2012) observed no significant alteration in TNF-α levels in MDD patients [53, 54]. Similarly, Myung et al. (2016) reported that TNF-α levels were not associated with MDD and found no significant correlation between Ham-D scores and TNF-α [55]. Another finding of our study was that we observed increased serum MCP-4 levels in MDD patients compared to HCs. However, the present study saw a negative correlation between elevated MCP-4 levels and the disease severity of MDD. Contrary to our results, Gao et al. (2022) observed that MCP-4 serum levels were significantly lowered in MDD patients (37.83±6.42 pg/ml) compared to HCs (98.25±8.04 pg/ml). In contrast to our observation, Gao et al. (2022) observed no significant correlation between Ham-D scores and MCP-4 serum levels. The observed discrepancy between elevated serum MCP-4 levels in MDD patients compared to HCs and a negative correlation between MCP-4 levels and Ham-D scores in MDD patients could have some potential explanations [32].

ROC analysis showed that serum TNF-α levels display good discriminatory value in differentiating MDD patients from HCs, evidenced by significantly higher AUC values (0.812) with 80.77% sensitivity and 73.58% specificity at a cut-off value of 31.78 pg/ml along with a diagnostic odd ratio of 11.71 and Youden’s index of 0.543. This result is in good agreement with the previously reported findings performed by Das et al. (2020), where TNF-α serum levels exhibited good predictive values with a significantly higher AUC of 0.866 and 81.60% sensitivity and 66.70% specificity at a cut-off value of 29.60 pg/ml [56]. Xu et al. (2023) also evaluated the diagnostic accuracy of TNF-α levels, and they reported serum TNF-α showed an AUC of 0.750 with 83.00% sensitivity and 59.00% specificity for potential use in predicting risk for developing MDD [18]. These findings indicate that the TNF-α levels might be used as a potential blood-based biomarker to evaluate the risk of developing MDD or to monitor the therapeutic response of antidepressants. However, serum MCP-4 levels showed moderate diagnostic efficacy in discriminating MDD patients from controls due to its lower percentages of sensitivity and specificity in diagnosing MDD patients. The calculated DOR displayed by this chemokine measurement was only 5.46 out of 10 as a good performance indicator. Also, Youden’s index was 0.394 out of 0.50 as the clinically effective discriminatory value. These calculated performance measures indicated that compared to TNF-α, MCP-4 serum levels exhibited lower efficacy in discriminating MDD patients from HCs. In contrast to our observation, Gao et al. (2022) reported that MCP-4 levels had good diagnostic value with an AUC value of 0.980 with 94.4% and 89.5% sensitivity and specificity, respectively at a cut-off value of 46.31 pg/ml where the lower value of MCP-4 resembles disease states [32].

Elevation of serum TNF-α levels in MDD patients can be reduced following the administration of antidepressants that potentiate the cytokine hypothesis in the pathogenesis of MDD [25, 27, 57]. Recently, some studies shed some light on the potential mechanism of action of TNF-α in depression. Zhu et al. (2006) showed that TNF-α stimulated serotonin (5-HT) uptake into presynaptic neurons by activating MAPK-mediated serotonin transporters to decrease the concentration of serotonin in the synaptic cleft [58]. As serotonin is one of the major neurotransmitters responsible for mood regulation, its reduced level leads to MDD. TNF-α enhances tryptophan metabolism by activating the indoleamine 2,3-dioxygenase enzyme, a catalyst for the conversion of tryptophan to kynurenine [59–60]. As tryptophan is the precursor for serotonin, TNF-α ultimately downregulates serotonin concentration by enhancing tryptophan depletion. TNF-α also activates the HPA axis associated with MDD pathophysiology [5]. Peripheral serum level of TNF-α is linked to neuro-inflammation as peripheral cytokines can cross the blood-brain barriers either through transporter protein-mediated active transport, transcellular migration, or through peripheral afferent nerves and can reach the CNS to activate microglia or other neuronal cells [60, 61]. Therefore, the transmigrated TNF-α can regulate the release of other pro-inflammatory cytokines such as IL-6, IL-1β, IL-8, etc. The secreted cytokines can activate neurodegenerative processes by triggering persistent chronic neuro-inflammatory activities such as excessive production of PGE2, reactive oxygen species (ROS), and reactive nitrogen species (RNS) [62]. All these mediators can damage neuronal circuits and pathways involved in mood regulation and thus lead to depressive symptoms.

Though there have been an increasing number of clinical and preclinical studies linking cytokines serum levels with the development of MDD, studies evaluating the role of chemokines such as MCP-4 are either inadequate or lacking [63, 64]. CC Chemokines trigger chemotaxis of monocyte-derived macrophages and other immune cells to the sites of infection and injured or inflamed tissues by influencing calcium influx mediated by the GPCR signaling pathway [65]. They also can contribute to inflammatory responses by inducing the secretion of pro-inflammatory mediators [63]. Chemokines can mediate the cross-talk between the peripheral immune system and the central nervous system by triggering the infiltration of leucocytes into sites of inflammation [15]. Elevated serum MCP-4 levels in MDD patients suggested an increased inflammatory or immune response in MDD. However, the negative correlation between MCP-4 and disease severity indicates that the role of MCP-4 in MDD is much more complex than was previously thought. The decreased MCP-4 levels in severely depressed patients might be due to the influence of one or more biological factors on MCP-4 levels that may cause the down-regulation of MCP-4 levels in patients with higher severity [30]. Another plausible explanation for this discrepancy was that inflammatory responses in MDD might display biphasic effects with low to moderate inflammatory activity. Therefore, lower MCP-4 levels might lead to disease development, and higher inflammatory response exhibited by higher MCP-4 levels reduces symptoms of MDD that can explain the elevated levels of MCP-4 in MDD patients with a negative correlation with disease severity [66]. Another probable explanation for these contradictory findings is that in this study population, MCP-4 might display both pro-inflammatory chemotactic activities leading to MDD development and neuroprotective functions diminishing the depressive symptoms. There might be a situation during the developmental phase of MDD where the pro-inflammatory chemotactic activity of MCP-4 was dominant to a certain level. Therefore, we saw elevated serum MCP-4 levels in MDD patients compared to HCs. These higher concentrations of MCP-4 might show more neuroprotective functions and other neuromodulatory effects that help to reduce disease severity. Madrigal et al. (2009) reported some neuroprotective functions of MCP-1 due to its ability to inhibit neuronal damage and block glutamate-mediated excitotoxicity [67]. Researchers found that MCP-1 has some neuromodulatory activities like stimulating neurogenesis, enhancing dopaminergic neurotransmission, etc. [65]. Proma et al. (2022) also observed a negative correlation between MCP-1 and the disease severity of MDD and suggested that the neuroprotective functions might be the reason [37]. As MCP-1 and MCP-4 belong to the same class of chemokines having similar biological activities [68], MCP-4 might display similar neuroprotective and dopaminergic activity-enhancing functions in CNS to improve symptoms of MDD. Therefore, further research is warranted to explore the underlying mechanisms of the complex role of MCP-4 in MDD. The heterogeneity of MDD might also explain this discrepancy. As MDD is not a single type of condition but a spectrum of different subtypes with various underlying factors, the observed negative correlation between MCP-4 levels and disease severity is only associated with this specific subtype of MDD patients in the Bangladeshi cohort. The observed discrepancy might also be due to the statistical noise stemming from the smaller sample size. Moreover, the weak negative correlation between Ham-D scores and MCP-4 levels (r = -0.27) indicates the requirement for further studies with a larger sample size and a more homogeneous population.

This case-control study evaluating the potential for serum TNF-α and MPC-4 levels as risk predictors for developing MDD in the Bangladeshi population has several clinical and therapeutic implications. Firstly, the study findings of elevated serum TNF-α levels in MDD patients compared to HCs and the significant and positive correlation between TNF-α levels and disease severity of MDD implicated that MDD patients of the Bangladeshi cohort displayed a higher level of inflammatory and immune responses. These findings support the cytokine hypothesis that pro-inflammatory cytokines are associated with MDD. Besides, we found a significant elevation of MCP-4 levels in MDD patients compared to HCs. However, a negative correlation between altered MCP-4 levels and disease severity indicates a complex role of MCP-4 in the pathophysiology of MDD. We hypothesized that plausible explanations for this negative correlation between MCP-4 levels and disease severity might be due to either the complexity of chemokine role in MDD in which other biological factors might downregulate MCP-4 levels in severely depressed patients or this might be similar to MCP-1, MCP-4 might display some neuroprotective and neuromodulatory effects. However, the significant elevation of MCP-4 in MDD patients compared to HCs indicated that MDD patients exhibited higher inflammatory responses than HCs. These findings will shed some light on our understanding of the role of cytokine and chemokine in MDD disease pathology. However, at this stage, we cannot conclude whether the altered serum TNF-α and MCP-4 levels are the etiological factors for the development of MDD or if they are just the outcomes of elevated inflammatory responses due to depressive episodes. More studies investigating the mechanism of actions of these cytokines in MDD disease pathophysiology are warranted. Recently, several clinical studies demonstrated that anti-inflammatory agents such as celecoxib, minocycline, TNF-α antagonists’ infliximab, and etanercept displayed some antidepressant activities or acted as adjunctive therapy in combination with antidepressants such as sertraline, fluoxetine by enhancing the efficacy of antidepressants [5, 69, 70]. Also, some studies showed that administration of currently available antidepressant drugs such as sertraline can reduce elevated baseline cytokines such as TNF-α or chemokine such as CCL2/MCP-1 [44, 65]. These results suggested that the TNF-α signaling pathway and MCP-4 signaling pathway could be a potential target for novel antidepressants or adjunctive therapy for antidepressants. In this study, we observed a significant association between TNF-α and MCP-4 and MDD disease severity that potentiated the cytokine hypothesis in MDD disease development and might pave the way for targeting TNF-α and MCP-4-mediated inflammatory pathways for the development of novel therapies against MDD.

Another principal implication of our study finding is that the increased serum TNF-α levels can evaluate the risk for developing MDD as TNF-α serum levels displayed a good diagnostic efficacy in discriminating MDD patients from HCs. The early prediction of developing MDD using serum TNF-α levels might be beneficial to start the management and therapeutic interventions for MDD at an early stage that can lower the rate of morbidity and suicidal tendency. Compared to other diagnostic tools, serum cytokine-based biomarkers are less invasive, more economical, and easier to perform. Moreover, cytokine-based biomarkers will exhibit an objective risk assessment compared to currently available subjective questionnaire-based detection tools. Thus, there is a growing interest among the scientific community in developing cytokine-based biomarkers for neuropsychiatric disorders like MDD. We thought that serum TNF-α levels have the potential to be used as a cytokine-based biomarker for the detection of MDD. However, further clinical and longitudinal studies are required to demonstrate the diagnostic efficacy of this cytokine.

Strengths of our study include the robust evidence supporting elevated serum TNF-α levels in MDD patients compared to HCs, supported by multiple studies with consistent findings. ROC analysis highlighted the diagnostic potential of serum TNF-α levels in discriminating MDD patients from HCs, suggesting a valuable biomarker for early risk assessment and monitoring therapeutic responses. Additionally, our study contributes to the growing body of evidence linking inflammation and depression, reinforcing the cytokine hypothesis of MDD pathogenesis. Furthermore, exploring potential mechanisms involving TNF-α, such as its impact on serotonin regulation, tryptophan metabolism, and the HPA axis, provides valuable insights into the complex relationship between cytokines and depression.

Nevertheless, it is imperative to acknowledge several constraints. Initially, the found disparity in MCP-4 levels and their inverse association with disease severity prompts inquiries on the involvement of MCP-4 in MDD, thus requiring additional research and replication in large and more homogeneous cohorts. The potential presence of statistical noise in the sample size, especially for HCs, suggests that obtaining more samples could yield more reliable and robust findings. Furthermore, it is vital to note that the case-control nature employed in this study imposes limitations on our capacity to show a causal relationship between modified cytokine levels and MDD. Longitudinal studies can thoroughly investigate this association over an extended period. Moreover, the observed discrepancy in cytokine expression between cohorts of Bangladeshi and Chinese individuals underscores the necessity of exercising prudence when extrapolating research findings to heterogeneous populations.

Despite these limitations, our study offers valuable clinical and therapeutic implications. The increased TNF-α levels in MDD patients compared to HCs support the cytokine theory of MDD pathophysiology. Additionally, the positive connection between TNF-α levels and disease severity implies a role for peripheral TNF-α in MDD pathophysiology. Serum TNF-α levels can distinguish MDD patients from controls, making it a valuable biomarker for early risk assessment and tracking therapy responses. Therefore, these findings could improve MDD detection and management, reducing disease burden. However, the role of MCP-4 in MDD is more complicated. Despite significantly increased blood MCP-4 levels in MDD patients, their negative connection with disease severity supports a diverse role for MCP-4 in MDD pathophysiology. However, this needs more study, primarily as chemokines like MCP-4 may protect and modulate the central nervous system. Ethnic heterogeneity in cytokine expression emphasizes the importance of population-specific factors in MDD inflammation research. Our findings suggest exploring new treatments for depression targeting TNF-α and MCP-4-mediated inflammatory pathways. To identify individuals at risk for MDD, cytokine-based biomarkers such as serum TNF-α levels may be a reliable and more objective approach. Our study advances our understanding of the complex relationship between inflammation and depression, promising better diagnosis and treatment of this debilitating mental health illness.

## 5. Conclusion

Our study highlights the complex connection between pro-inflammatory cytokines, such as TNF-α, and MDD. The potential for serum TNF-α levels as a biomarker for MDD risk assessment and monitoring therapeutic responses could have significant clinical utility, particularly for early intervention. Additionally, our findings reinforce the potential for targeting TNF-α and MCP-4-mediated inflammatory pathways to identify novel antidepressant therapies. Overall, our study contributes to the evolving understanding of the relationship between inflammation and depression, paving the way for further research in this critical area of mental health. However, we recommend further research on large and homogenous populations and longitudinal studies to demonstrate causality and corroborate these findings.

## Supporting information

S1 Checklist STROBE statement—checklist of items that should be included in reports of observational studies. (DOCX)

## References

[1] Salsabil L, Shahriar M, Islam SMA, Bhuiyan MA, Qusar MS, Islam MR. Higher serum nerve growth factor levels are associated with major depressive disorder pathophysiology: a case-control study. J Int Med Res. 2023;51(4):3000605231166222. doi: 10.1177/03000605231166222 37038918 PMC10107982

[2] Nishuty NL, Khandoker MMH, Karmoker JR, et al. Evaluation of Serum Interleukin-6 and C-reactive Protein Levels in Drug-naïve Major Depressive Disorder Patients. Cureus. 2019;11(1):e3868. Published 2019 Jan 11. doi: 10.7759/cureus.3868 30899619 PMC6414189

[3] Rahman S, Shanta AA, Daria S, et al. Increased serum resistin but not G-CSF levels are associated in the pathophysiology of major depressive disorder: Findings from a case-control study. PLoS One. 2022;17(2):e0264404. Published 2022 Feb 25. doi: 10.1371/journal.pone.0264404 35213631 PMC8880862

[4] Möller HJ. Suicide, suicidality and suicide prevention in affective disorders. Acta Psychiatr Scand Suppl. 2003;(418):73–80. . 12956819

[5] Ma K, Zhang H, Baloch Z. Pathogenetic and Therapeutic Applications of Tumor Necrosis Factor-α (TNF-α) in Major Depressive Disorder: A Systematic Review. Int J Mol Sci. 2016;17(5):733. Published 2016 May 14. doi: 10.3390/ijms17050733 27187381 PMC4881555

[6] Labermaier C, Masana M, Müller MB. Biomarkers predicting antidepressant treatment response: how can we advance the field? Dis Markers. 2013;35(1):23–31. doi: 10.1155/2013/984845 Epub 2013 Jul 21. ; PMCID: PMC3774965. 24167346 PMC3774965

[7] Emon MPZ, Das R, Nishuty NL, Shalahuddin Qusar MMA, Bhuiyan MA, Islam MR. Reduced serum BDNF levels are associated with the increased risk for developing MDD: a case-control study with or without antidepressant therapy. BMC Res Notes. 2020;13(1):83. Published 2020 Feb 21. doi: 10.1186/s13104-020-04952-3 32085720 PMC7035767

[8] Bains N, Abdijadid S. Major Depressive Disorder. In: StatPearls. Treasure Island (FL): StatPearls Publishing; April 10, 2023. 34033316

[9] Teleanu RI, Niculescu AG, Roza E, Vladâcenco O, Grumezescu AM, Teleanu DM. Neurotransmitters-Key Factors in Neurological and Neurodegenerative Disorders of the Central Nervous System. Int J Mol Sci. 2022;23(11):5954. Published 2022 May 25. doi: 10.3390/ijms23115954 35682631 PMC9180936

[10] Otte C, Gold SM, Penninx BW, et al. Major depressive disorder. Nat Rev Dis Primers. 2016;2:16065. Published 2016 Sep 15. doi: 10.1038/nrdp.2016.65 27629598

[11] Tian H, Hu Z, Xu J, Wang C. The molecular pathophysiology of depression and the new therapeutics. MedComm (2020). 2022;3(3):e156. Published 2022 Jul 21. doi: 10.1002/mco2.156 35875370 PMC9301929

[12] Gałecki P, Talarowska M. Inflammatory theory of depression. Teoria zapalna depresji–najważniejsze fakty. Psychiatr Pol. 2018;52(3):437–447. doi: 10.12740/PP/76863 30218560

[13] Daria S, Proma MA, Shahriar M, Islam SMA, Bhuiyan MA, Islam MR. Serum interferon-gamma level is associated with drug-naïve major depressive disorder. SAGE Open Med. 2020;8:2050312120974169. Published 2020 Nov 20. doi: 10.1177/2050312120974169 33282305 PMC7682211

[14] Nahar Z, Monisha ST, Qusar MS, Islam MR. Evaluation of serum interleukin-1 receptor antagonist levels in major depressive disorder: A case-control study. Health Sci Rep. 2023;6(4):e1175. Published 2023 Mar 29. doi: 10.1002/hsr2.1175 37008817 PMC10050969

[15] Milenkovic VM, Sarubin N, Hilbert S, et al. Macrophage-Derived Chemokine: A Putative Marker of Pharmacological Therapy Response in Major Depression?. Neuroimmunomodulation. 2017;24(2):106–112. doi: 10.1159/000479739 28898872

[16] Islam MR, Sohan M, Daria S, Masud AA, Ahmed MU, Roy A, et al. Evaluation of inflammatory cytokines in drug-naïve major depressive disorder: A systematic review and meta-analysis. Int J Immunopathol Pharmacol. 2023 Jan-Dec;37:3946320231198828. doi: 10.1177/03946320231198828 ; PMCID: PMC10467201. 37625799 PMC10467201

[17] Pitsillou E, Bresnehan SM, Kagarakis EA, et al. The cellular and molecular basis of major depressive disorder: towards a unified model for understanding clinical depression. Mol Biol Rep. 2020;47(1):753–770. doi: 10.1007/s11033-019-05129-3 31612411

[18] Xu Y, Liang J, Sun Y, Zhang Y, Shan F, Ge J, et al. Serum cytokines-based biomarkers in the diagnosis and monitoring of therapeutic response in patients with major depressive disorder. Int Immunopharmacol. 2023 May;118:110108. doi: 10.1016/j.intimp.2023.110108 Epub 2023 Mar 31. . 37004349

[19] Leighton SP, Nerurkar L, Krishnadas R, Johnman C, Graham GJ, Cavanagh J. Chemokines in depression in health and in inflammatory illness: a systematic review and meta-analysis. Mol Psychiatry. 2018 Jan;23(1):48–58. doi: 10.1038/mp.2017.205 Epub 2017 Nov 14. ; PMCID: PMC5754468. 29133955 PMC5754468

[20] Eyre HA, Air T, Pradhan A, Johnston J, Lavretsky H, Stuart MJ, et al. A meta-analysis of chemokines in major depression. Prog Neuropsychopharmacol Biol Psychiatry. 2016 Jul 4;68:1–8. doi: 10.1016/j.pnpbp.2016.02.006 Epub 2016 Feb 20. ; PMCID: PMC5536843. 26903140 PMC5536843

[21] de la Peña FR, Cruz-Fuentes C, Palacios L, Girón-Pérez MI, Medina-Rivero E, Ponce-Regalado MD, et al. Serum levels of chemokines in adolescents with major depression treated with fluoxetine. World J Psychiatry. 2020 Aug 19;10(8):175–186. doi: 10.5498/wjp.v10.i8.175 ; PMCID: PMC7439300. 32874955 PMC7439300

[22] Nahar Z, Sal-Sabil N, Sohan M, Qusar MS, Islam MR. Higher serum interleukin-12 levels are associated with the pathophysiology of major depressive disorder: A case-control study results. Health Sci Rep. 2022 Dec 24;6(1):e1005. doi: 10.1002/hsr2.1005 ; PMCID: PMC9789678. 36582626 PMC9789678

[23] Osimo EF, Pillinger T, Rodriguez IM, Khandaker GM, Pariante CM, Howes OD. Inflammatory markers in depression: A meta-analysis of mean differences and variability in 5,166 patients and 5,083 controls. Brain Behav Immun. 2020;87:901–909. doi: 10.1016/j.bbi.2020.02.010 32113908 PMC7327519

[24] Carvalho AF, Solmi M, Sanches M, et al. Evidence-based umbrella review of 162 peripheral biomarkers for major mental disorders. Transl Psychiatry. 2020;10(1):152. Published 2020 May 18. doi: 10.1038/s41398-020-0835-5 32424116 PMC7235270

[25] Dowlati Y, Herrmann N, Swardfager W, et al. A meta-analysis of cytokines in major depression. Biol Psychiatry. 2010;67(5):446–457. doi: 10.1016/j.biopsych.2009.09.033 20015486

[26] Liu Y, Ho RC, Mak A. Interleukin (IL)-6, tumour necrosis factor alpha (TNF-α) and soluble interleukin-2 receptors (sIL-2R) are elevated in patients with major depressive disorder: a meta-analysis and meta-regression. J Affect Disord. 2012;139(3):230–239. doi: 10.1016/j.jad.2011.08.003 21872339

[27] Köhler CA, Freitas TH, Maes M, et al. Peripheral cytokine and chemokine alterations in depression: a meta-analysis of 82 studies. Acta Psychiatr Scand. 2017;135(5):373–387. doi: 10.1111/acps.12698 28122130

[28] Strawbridge R, Arnone D, Danese A, Papadopoulos A, Herane Vives A, Cleare AJ. Inflammation and clinical response to treatment in depression: A meta-analysis. Eur Neuropsychopharmacol. 2015;25(10):1532–1543. doi: 10.1016/j.euroneuro.2015.06.007 26169573

[29] Nobis A, Zalewski D, Waszkiewicz N. Peripheral Markers of Depression. J Clin Med. 2020;9(12):3793. Published 2020 Nov 24. doi: 10.3390/jcm9123793 33255237 PMC7760788

[30] Goldsmith DR, Rapaport MH, Miller BJ. A meta-analysis of blood cytokine network alterations in psychiatric patients: comparisons between schizophrenia, bipolar disorder and depression. Mol Psychiatry. 2016;21(12):1696–1709. doi: 10.1038/mp.2016.3 26903267 PMC6056174

[31] Dalgard C, Eidelman O, Jozwik C, et al. The MCP-4/MCP-1 ratio in plasma is a candidate circadian biomarker for chronic post-traumatic stress disorder. Transl Psychiatry. 2017;7(2):e1025. Published 2017 Feb 7. doi: 10.1038/tp.2016.285 28170001 PMC5438024

[32] Gao W, Xu Y, Liang J, Sun Y, Zhang Y, Shan F, et al. Serum CC Chemokines as Potential Biomarkers for the Diagnosis of Major Depressive Disorder. Psychol Res Behav Manag. 2022 Oct 11;15:2971–2978. doi: 10.2147/PRBM.S384267 ; PMCID: PMC9604417. 36310625 PMC9604417

[33] Garcia-Zepeda EA, Combadiere C, Rothenberg ME, et al. Human monocyte chemoattractant protein (MCP)-4 is a novel CC chemokine with activities on monocytes, eosinophils, and basophils induced in allergic and nonallergic inflammation that signals through the CC chemokine receptors (CCR)-2 and -3. J Immunol. 1996;157(12):5613–5626. 8955214

[34] Feiger JA, Snyder RL, Walsh MJ, et al. The Role of Neuroinflammation in Neuropsychiatric Disorders Following Traumatic Brain Injury: A Systematic Review. J Head Trauma Rehabil. 2022;37(5):E370–E382. doi: 10.1097/HTR.0000000000000754 35125427

[35] Furtado M, Katzman MA. Examining the role of neuroinflammation in major depression. Psychiatry Res. 2015;229(1–2):27–36. doi: 10.1016/j.psychres.2015.06.009 26187338

[36] Islam S, Islam T, Nahar Z, Shahriar M, Islam SMA, Bhuiyan MA, et al. Altered serum adiponectin and interleukin-8 levels are associated in the pathophysiology of major depressive disorder: A case-control study. PLoS One. 2022 Nov 21;17(11):e0276619. doi: 10.1371/journal.pone.0276619 ; PMCID: PMC9678262. 36409748 PMC9678262

[37] Proma MA, Daria S, Nahar Z, Ashraful Islam SM, Bhuiyan MA, Islam MR. Monocyte chemoattractant protein-1 levels are associated with major depressive disorder. J Basic Clin Physiol Pharmacol. 2022 Jan 5;33(6):735–741. doi: 10.1515/jbcpp-2021-0132 . 34983131

[38] Riya S, Sultana S, Daria S, Proma MA, Bhuiyan MA, Haque MA, et al. Evaluation of Serum Lysophosphatidic Acid and Lysophosphatidylcholine Levels in Major Depressive Disorder Patients. Cureus. 2020 Dec 30;12(12):e12388. doi: 10.7759/cureus.12388 ; PMCID: PMC7849208. 33542861 PMC7849208

[39] Ali S, Nahar Z, Rahman MR, Islam SMA, Bhuiyan MA, Islam MR. Serum insulin-like growth factor-1 and relaxin-3 are linked with major depressive disorder. Asian J Psychiatr. 2020;53:102164. doi: 10.1016/j.ajp.2020.102164 32446216

[40] Islam MR, Ali S, Karmoker JR, et al. Evaluation of serum amino acids and non-enzymatic antioxidants in drug-naïve first-episode major depressive disorder. BMC Psychiatry. 2020;20(1):333. Published 2020 Jun 24. doi: 10.1186/s12888-020-02738-2 32580709 PMC7315550

[41] Anjum S, Qusar MMAS, Shahriar M, Islam SMA, i MA Islam MR. Altered serum interleukin-7 and interleukin-10 are associated with drug-free major depressive disorder. Ther Adv Psychopharmacol. 2020;10:2045125320916655. Published 2020 Apr 28. doi: 10.1177/2045125320916655 32435448 PMC7225792

[42] Islam MR, Islam MR, Shalahuddin Qusar MMA, et al. Alterations of serum macro-minerals and trace elements are associated with major depressive disorder: a case-control study. BMC Psychiatry. 2018;18(1):94. Published 2018 Apr 10. doi: 10.1186/s12888-018-1685-z 29631563 PMC5891975

[43] Fan N, Luo Y, Ou Y, He H. Altered serum levels of TNF-α, IL-6, and IL-18 in depressive disorder patients. Hum Psychopharmacol. 2017;32(4):10.1002/hup.2588. doi: 10.1002/hup.2588 28582802

[44] Yao L, Pan L, Qian M, et al. Tumor Necrosis Factor-α Variations in Patients With Major Depressive Disorder Before and After Antidepressant Treatment. Front Psychiatry. 2020;11:518837. Published 2020 Dec 7. doi: 10.3389/fpsyt.2020.518837 33364982 PMC7750423

[45] Chen Y, Ouyang J, Liu S, Zhang S, Chen P, Jiang T. The Role of Cytokines in the Peripheral Blood of Major Depressive Patients. Clin Lab. 2017 Jul 1;63(7):1207–1212. doi: 10.7754/Clin.Lab.2017.170117 . 28792696

[46] Al-Hakeim HK, Al-Rammahi DA, Al-Dujaili AH. IL-6, IL–18, sIL-2R, and TNFα proinflammatory markers in depression and schizophrenia patients who are free of overt inflammation. J Affect Disord. 2015 Aug 15;182:106–14. doi: 10.1016/j.jad.2015.04.044 Epub 2015 May 5. . 25985379

[47] Min X, Wang G, Cui Y, Meng P, Hu X, Liu S, et al. Association between inflammatory cytokines and symptoms of major depressive disorder in adults. Front Immunol. 2023 Feb 13;14:1110775. doi: 10.3389/fimmu.2023.1110775 ; PMCID: PMC9968963. 36860860 PMC9968963

[48] Mikova O, Yakimova R, Bosmans E, Kenis G, Maes M. Increased serum tumor necrosis factor alpha concentrations in major depression and multiple sclerosis. Eur Neuropsychopharmacol. 2001 Jun;11(3):203–8. doi: 10.1016/s0924-977x(01)00081-5 . 11418279

[49] Tuglu C, Kara SH, Caliyurt O, Vardar E, Abay E. Increased serum tumor necrosis factor-alpha levels and treatment response in major depressive disorder. Psychopharmacology (Berl). 2003 Dec;170(4):429–33. doi: 10.1007/s00213-003-1566-z Epub 2003 Aug 30. . 12955291

[50] Yang K, Xie G, Zhang Z, Wang C, Li W, Zhou W, et al. Levels of serum interleukin (IL)-6, IL-1beta, tumour necrosis factor-alpha and leptin and their correlation in depression. Aust N Z J Psychiatry. 2007 Mar;41(3):266–73. doi: 10.1080/00048670601057759 . 17464708

[51] Zou W, Feng R, Yang Y. Changes in the serum levels of inflammatory cytokines in antidepressant drug-naïve patients with major depression. PLoS One. 2018 Jun 1;13(6):e0197267. doi: 10.1371/journal.pone.0197267 ; PMCID: PMC5983476. 29856741 PMC5983476

[52] Leo R, Di Lorenzo G, Tesauro M, et al. Association between enhanced soluble CD40 ligand and proinflammatory and prothrombotic states in major depressive disorder: pilot observations on the effects of selective serotonin reuptake inhibitor therapy. J Clin Psychiatry. 2006;67(11):1760–1766. doi: 10.4088/jcp.v67n1114 17196057

[53] Farid Hosseini R, Jabbari Azad F, Talaee A, et al. Assessment of the immune system activity in Iranian patients with Major Depression Disorder (MDD). Iran J Immunol. 2007;4(1):38–43. 17652842 10.22034/iji.2007.17178

[54] Einvik G, Vistnes M, Hrubos-Strøm H, et al. Circulating cytokine concentrations are not associated with major depressive disorder in a community-based cohort. Gen Hosp Psychiatry. 2012;34(3):262–267. doi: 10.1016/j.genhosppsych.2012.01.017 22401706

[55] Myung W, Lim SW, Woo HI, et al. Serum Cytokine Levels in Major Depressive Disorder and Its Role in Antidepressant Response. Psychiatry Investig. 2016;13(6):644–651. doi: 10.4306/pi.2016.13.6.644 27909456 PMC5128353

[56] Das R, Emon MPZ, Shahriar M, Nahar Z, Islam SMA, Bhuiyan MA, et al. Higher levels of serum IL-1β and TNF-α are associated with an increased probability of major depressive disorder. Psychiatry Res. 2021 Jan;295:113568. doi: 10.1016/j.psychres.2020.113568 Epub 2020 Nov 10. . 33199026

[57] Liu JJ, Wei YB, Strawbridge R, Bao Y, Chang S, Shi L, et al. Peripheral cytokine levels and response to antidepressant treatment in depression: a systematic review and meta-analysis. Mol Psychiatry. 2020 Feb;25(2):339–350. doi: 10.1038/s41380-019-0474-5 Epub 2019 Aug 19. . 31427752

[58] Zhu CB, Blakely RD, Hewlett WA. The proinflammatory cytokines interleukin-1beta and tumor necrosis factor-alpha activate serotonin transporters. Neuropsychopharmacology. 2006 Oct;31(10):2121–31. doi: 10.1038/sj.npp.1301029 Epub 2006 Feb 1. . 16452991

[59] Wichers M, Maes M. The psychoneuroimmuno-pathophysiology of cytokine-induced depression in humans. Int J Neuropsychopharmacol. 2002 Dec;5(4):375–88. doi: 10.1017/S1461145702003103 . 12466036

[60] Miller AH, Raison CL. The role of inflammation in depression: from evolutionary imperative to modern treatment target. Nat Rev Immunol. 2016 Jan;16(1):22–34. doi: 10.1038/nri.2015.5 ; PMCID: PMC5542678. 26711676 PMC5542678

[61] Miller AH, Maletic V, Raison CL. Inflammation and its discontents: the role of cytokines in the pathophysiology of major depression. Biol Psychiatry. 2009 May 1;65(9):732–41. doi: 10.1016/j.biopsych.2008.11.029 Epub 2009 Jan 15. ; PMCID: PMC2680424. 19150053 PMC2680424

[62] Kempuraj D, Thangavel R, Natteru PA, Selvakumar GP, Saeed D, Zahoor H, et al. Neuroinflammation Induces Neurodegeneration. J Neurol Neurosurg Spine. 2016;1(1):1003. Epub 2016 Nov 18. ; PMCID: PMC5260818. 28127589 PMC5260818

[63] Stuart MJ, Singhal G, Baune BT. Systematic Review of the Neurobiological Relevance of Chemokines to Psychiatric Disorders. Front Cell Neurosci. 2015 Sep 10;9:357. doi: 10.3389/fncel.2015.00357 ; PMCID: PMC4564736. 26441528 PMC4564736

[64] Stuart MJ, Baune BT. Chemokines and chemokine receptors in mood disorders, schizophrenia, and cognitive impairment: a systematic review of biomarker studies. Neurosci Biobehav Rev. 2014 May;42:93–115. doi: 10.1016/j.neubiorev.2014.02.001 Epub 2014 Feb 8. . 24513303

[65] Curzytek K, Leśkiewicz M. Targeting the CCL2-CCR2 axis in depressive disorders. Pharmacol Rep. 2021 Aug;73(4):1052–1062. doi: 10.1007/s43440-021-00280-w Epub 2021 May 24. ; PMCID: PMC8142870. 34031863 PMC8142870

[66] DeForge LE, Kenney JS, Jones ML, Warren JS, Remick DG. Biphasic production of IL-8 in lipopolysaccharide (LPS)-stimulated human whole blood. Separation of LPS- and cytokine-stimulated components using anti-tumor necrosis factor and anti-IL-1 antibodies. J Immunol. 1992;148(7):2133–2141. 1545121

[67] Madrigal JL, Leza JC, Polak P, Kalinin S, Feinstein DL. Astrocyte-derived MCP-1 mediates neuroprotective effects of noradrenaline. J Neurosci. 2009 Jan 7;29(1):263–7. doi: 10.1523/JNEUROSCI.4926-08.2009 ; PMCID: PMC6664914. 19129402 PMC6664914

[68] Benarafa C, Cunningham FM, Hamblin AS, Horohov DW, Collins ME. Cloning of equine chemokines eotaxin, monocyte chemoattractant protein (MCP)-1, MCP-2 and MCP-4, mRNA expression in tissues and induction by IL-4 in dermal fibroblasts. Vet Immunol Immunopathol. 2000;76(3–4):283–298. doi: 10.1016/s0165-2427(00)00222-1 11044560

[69] Leonard BE. Inflammation and depression: a causal or coincidental link to the pathophysiology? Acta Neuropsychiatr. 2018 Feb;30(1):1–16. doi: 10.1017/neu.2016.69 Epub 2017 Jan 23. . 28112061

[70] Abbasi SH, Hosseini F, Modabbernia A, Ashrafi M, Akhondzadeh S. Effect of celecoxib add-on treatment on symptoms and serum IL-6 concentrations in patients with major depressive disorder: randomized double-blind placebo-controlled study. J Affect Disord. 2012;141:308–314. doi: 10.1016/j.jad.2012.03.033 22516310

